# Supplementary material for: Design, Synthesis, and Evaluation of Monoamine Oxidase A Inhibitors–Indocyanine Dyes Conjugates as Targeted Antitumor Agents
Source: Molecules. 2019 Apr 10;24(7):1400. doi: 10.3390/molecules24071400 (PMC6480602; doi:10.3390/molecules24071400)
Supplement: Supplementary file 1 [file molecules-24-01400-s001.pdf]

Supplementary Materials

Design, synthesis, and evaluation of potential monoamine oxidase A inhibitors-indocyanine dyes conjugates as targeted antitumor agents

Xiao-Guang Yang [1,†], Yan-Hua Mou [2,†], Yong-Jun Wang [3], Jian Wang [1], Yan-Yu Li [1], Rui-Heng Kong [1], Meng Ding [1], Dun Wang [1,*], Chun Guo [1,*]

1. Key Laboratory of Structure-Based Drug Design & Discovery of Ministry of Education, Shenyang Pharmaceutical University, Shenyang 110016, China; xiaog_yang@163.com (X.-G.Y.); jianwang@syphu.edu.cn (J.W); 18345384016@163.com (Y.-Y.L.); 1157934775@163.com (R.-H.K.); dingmeng74376@126.com (M.D.)
2. School of life sciences and biological pharmacy, Shenyang Pharmaceutical University, Shenyang 110016, China; mouyanhua2018@163.com (Y.-H.M.)
3. Wuya College of innovation, Shenyang Pharmaceutical University, Shenyang110016, China; i_maple@163.com (Y.-J.W.)

* Correspondence: chunguo@syphu.edu.cn (C.G); wangduncn@163.com (D.W)

† These authors contributed equally.

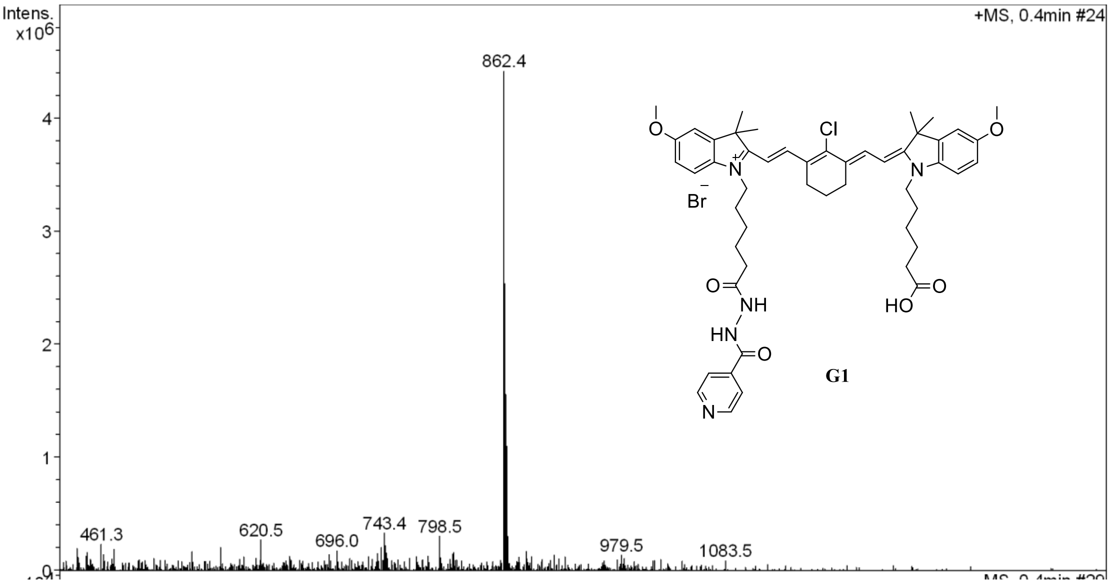

Fig. 1 MS spectrum of compound G1

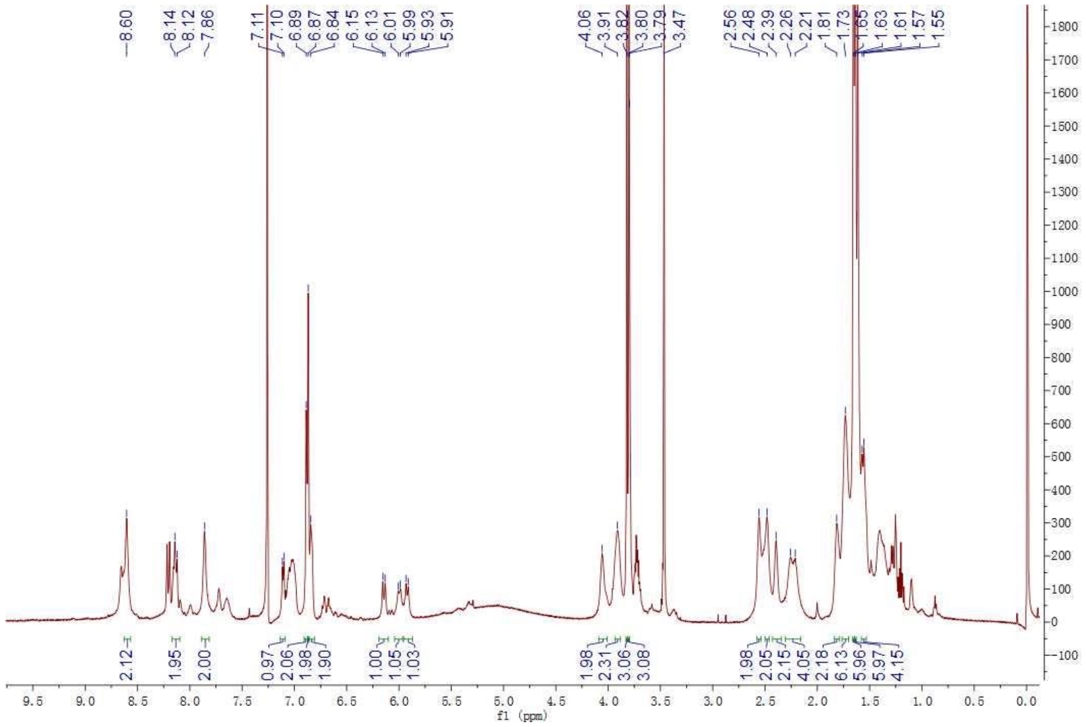

Fig. 2 ^{1}H-NMR spectrum of compound G1

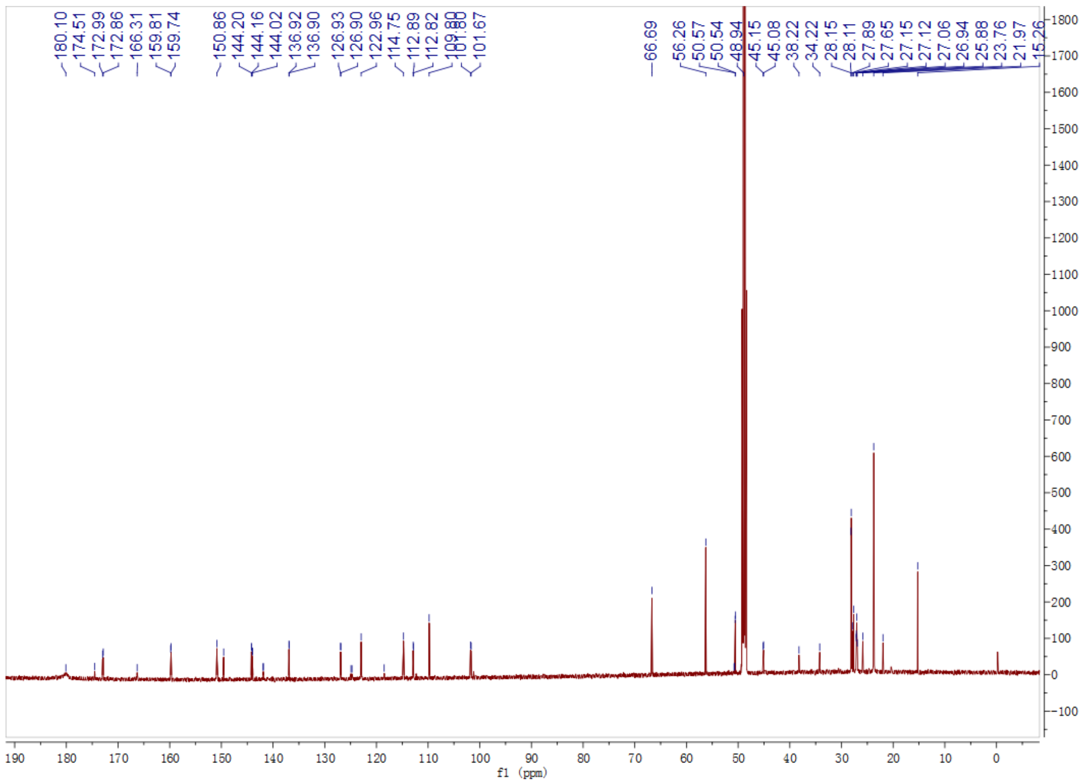

Fig. 3 ^{13}C-NMR spectrum of compound G1

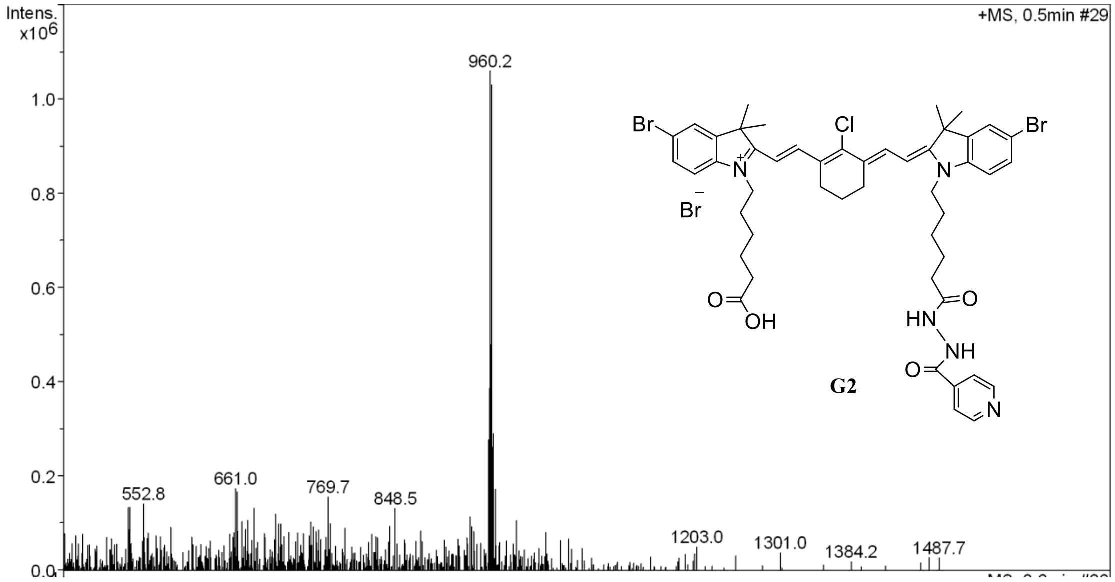

Fig. 4 MS spectrum of compound G2

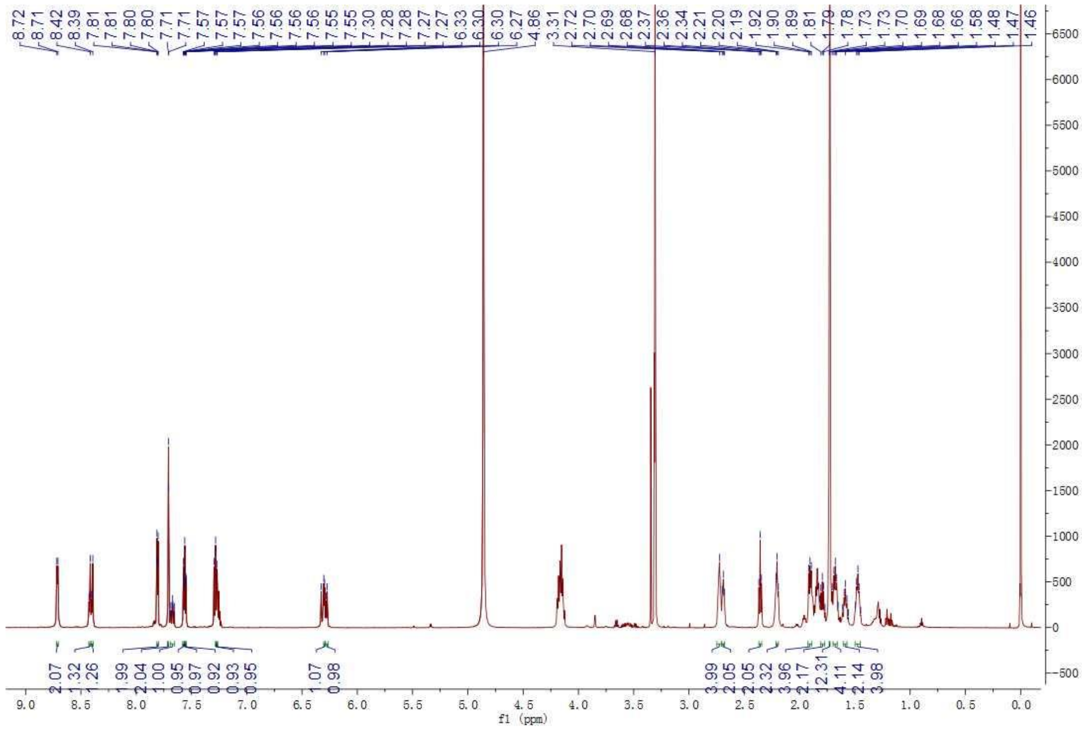

Fig. 5 ^{1}H-NMR spectrum of compound G2

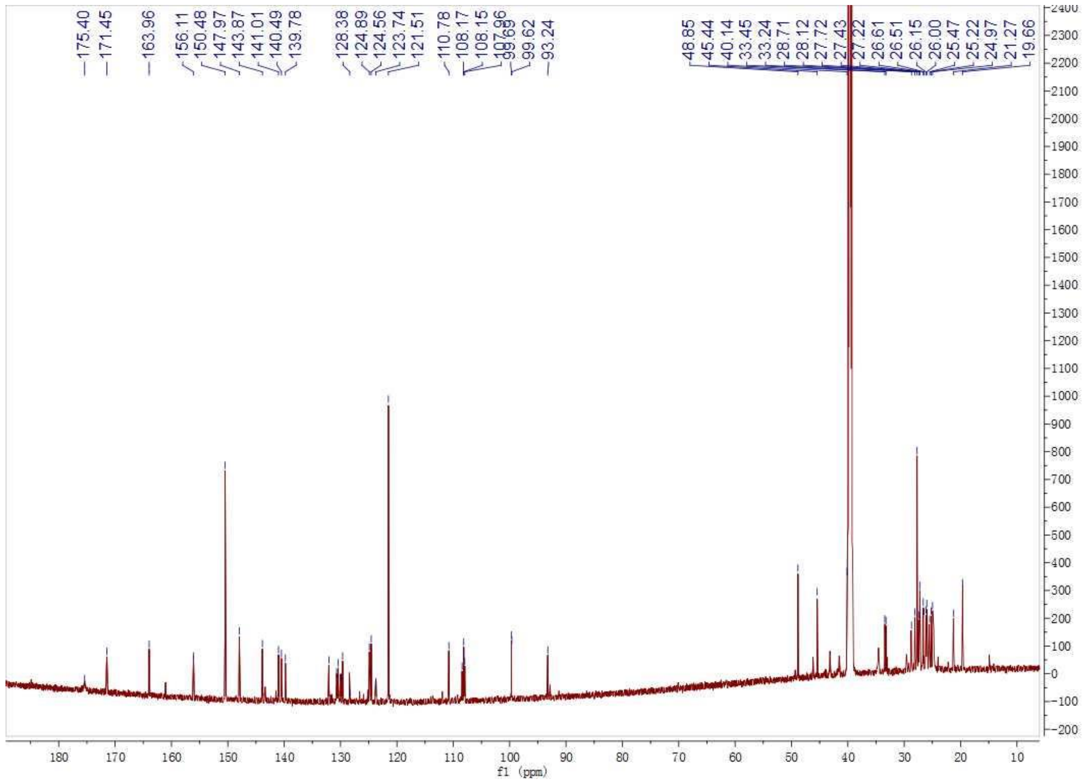

Fig. 6 ^{13}C-NMR spectrum of compound G2

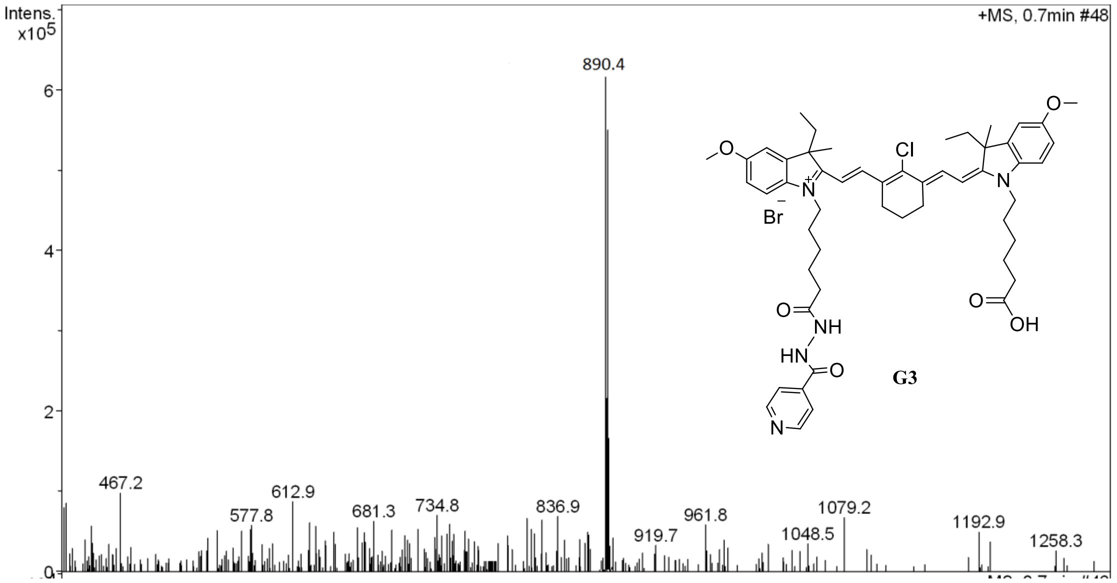

Fig. 7 MS spectrum of compound G3

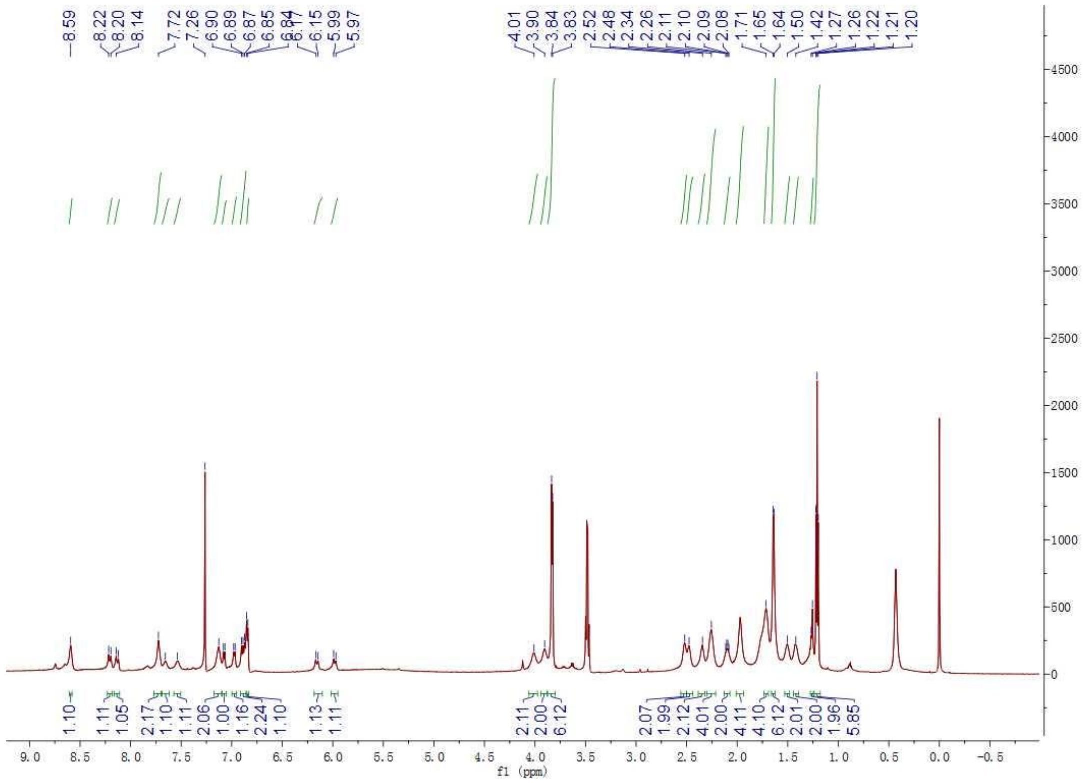

Fig. 8 ^{1}H-NMR spectrum of compound G3

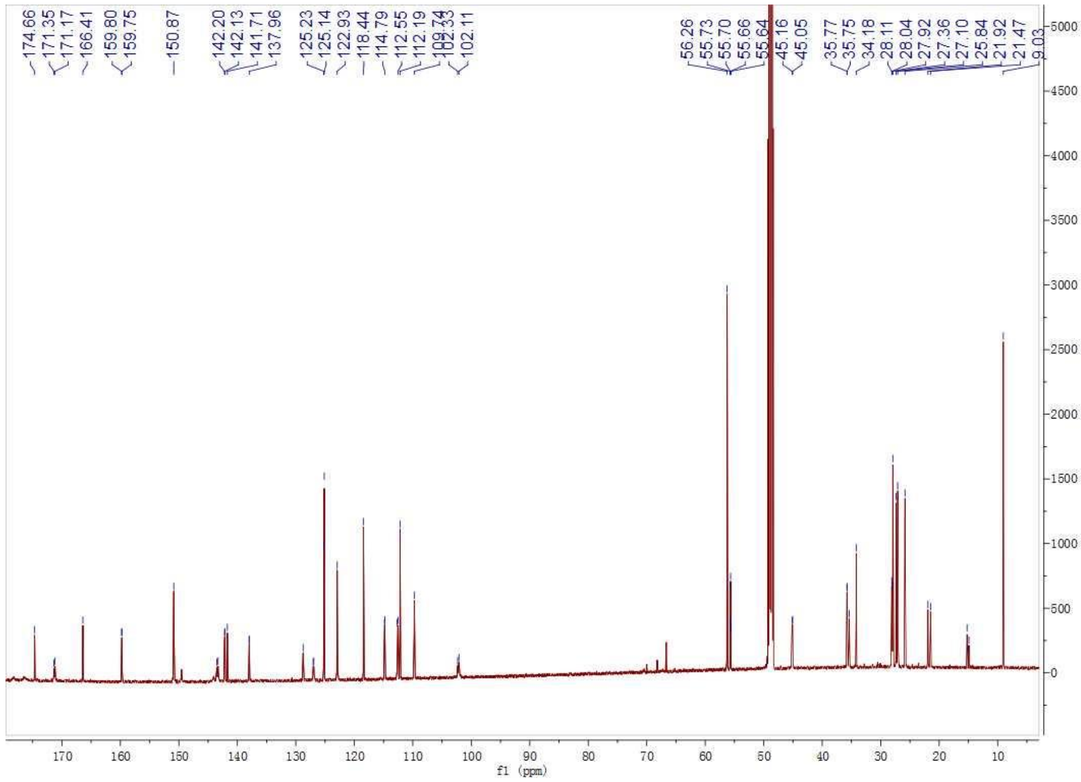

Fig. 9 ^{13}C-NMR spectrum of compound G3

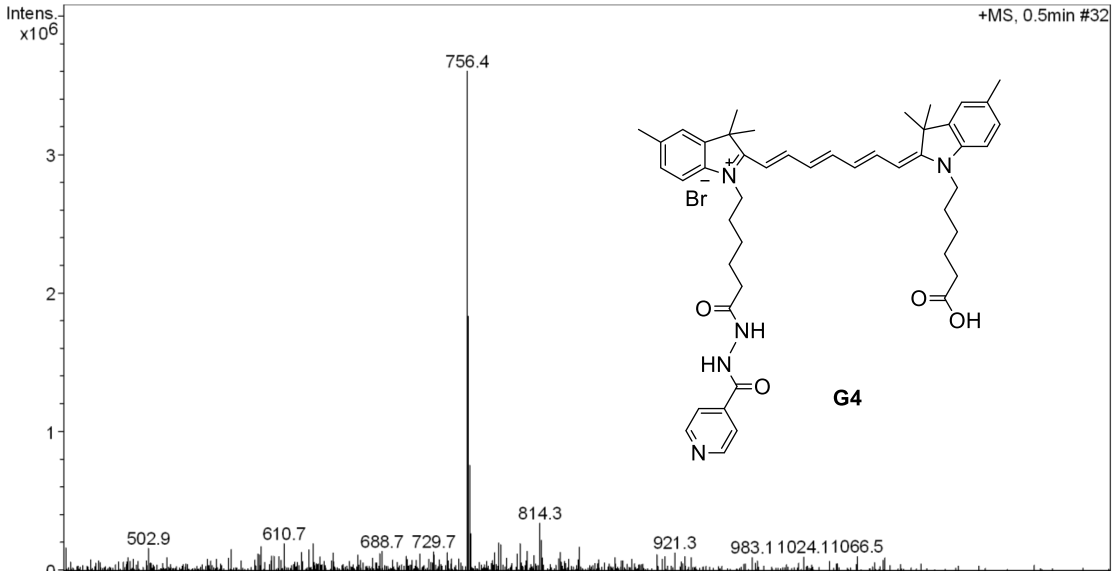

Fig. 10 MS spectrum of compound G4

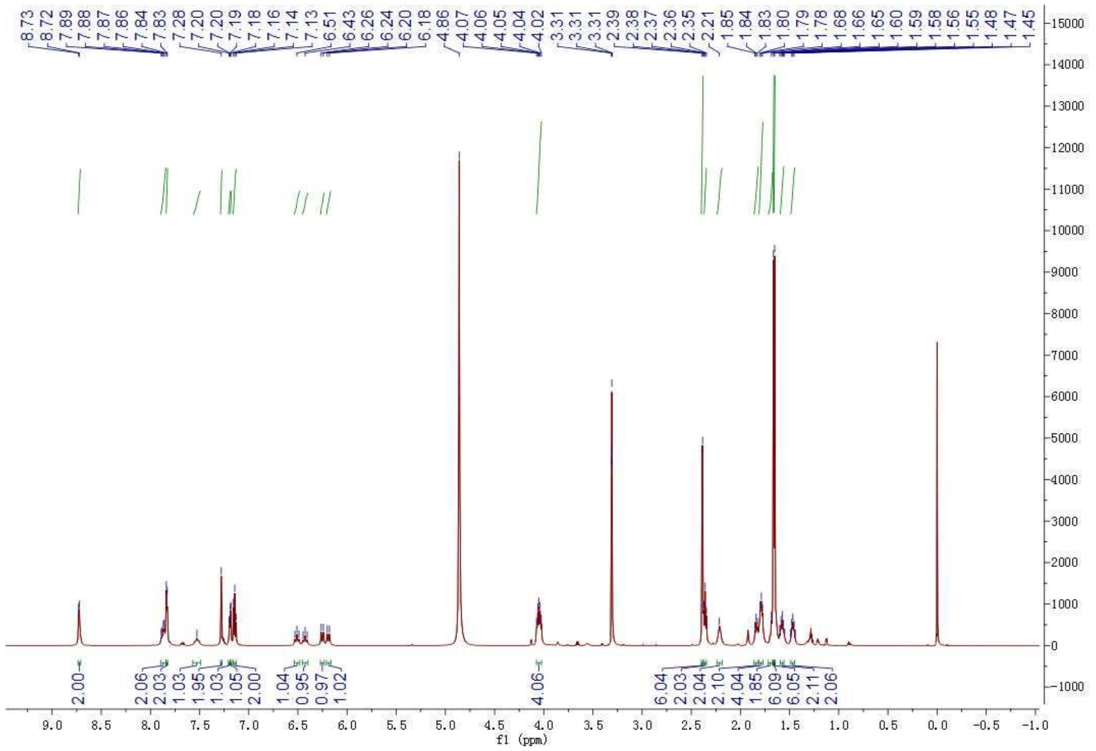

Fig. 11 ¹H-NMR spectrum of compound G4

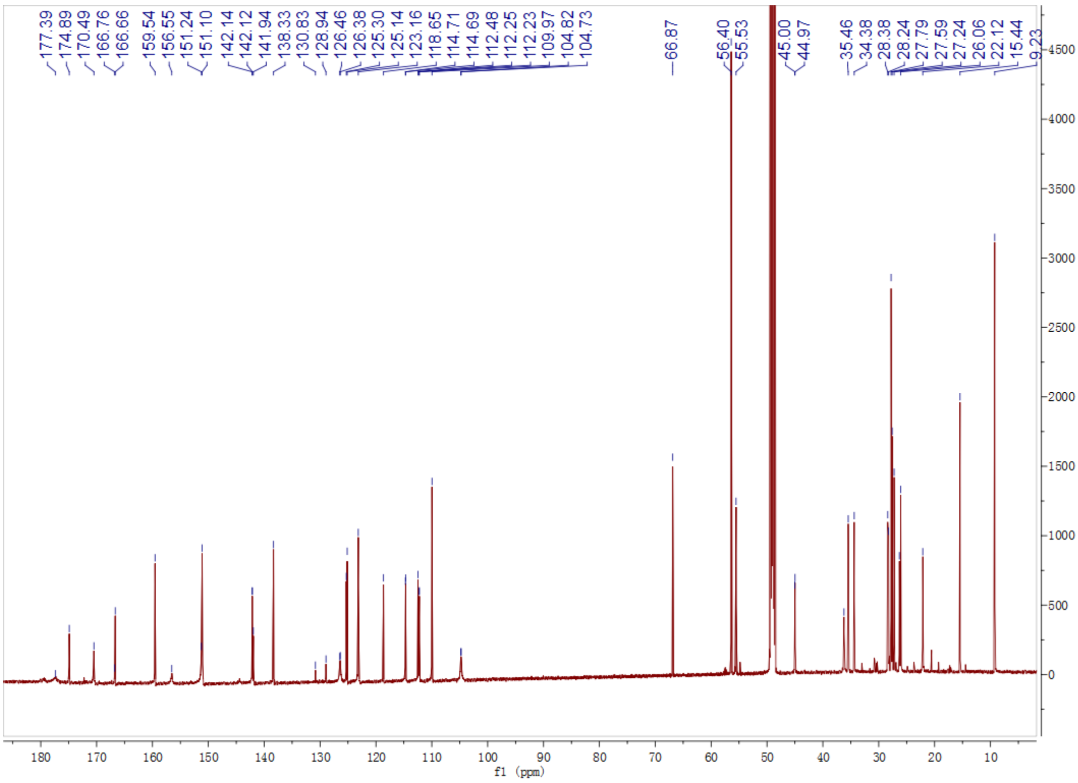

Fig. 12 ^{13}C-NMR spectrum of compound G4

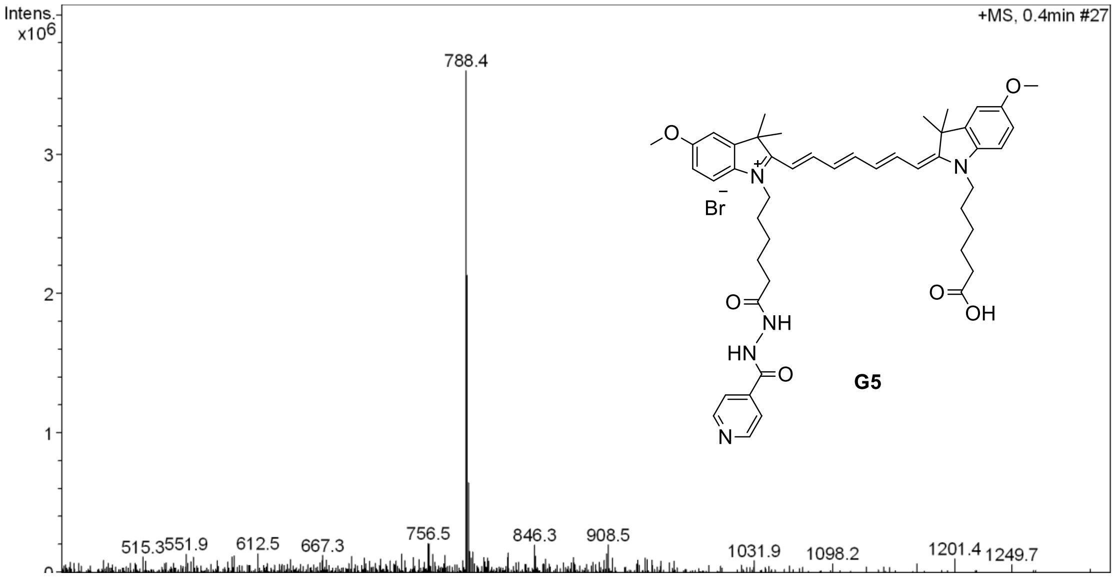

Fig. 13 MS spectrum of compound G5

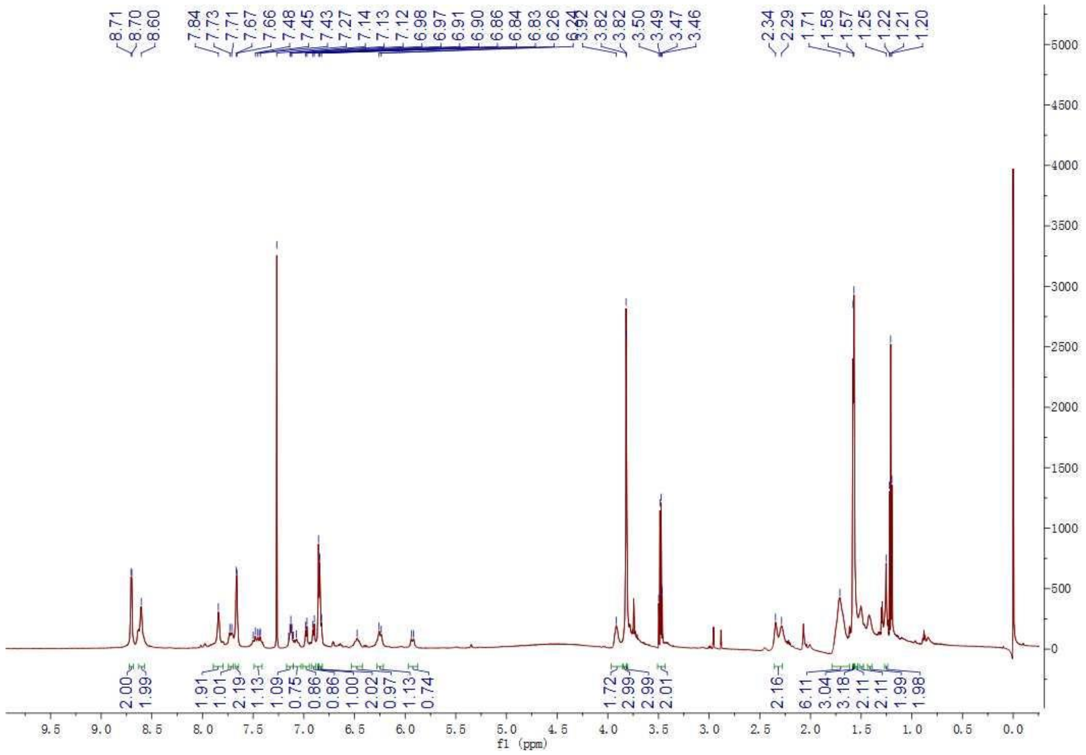

Fig. 14 ^{1}H-NMR spectrum of compound G5

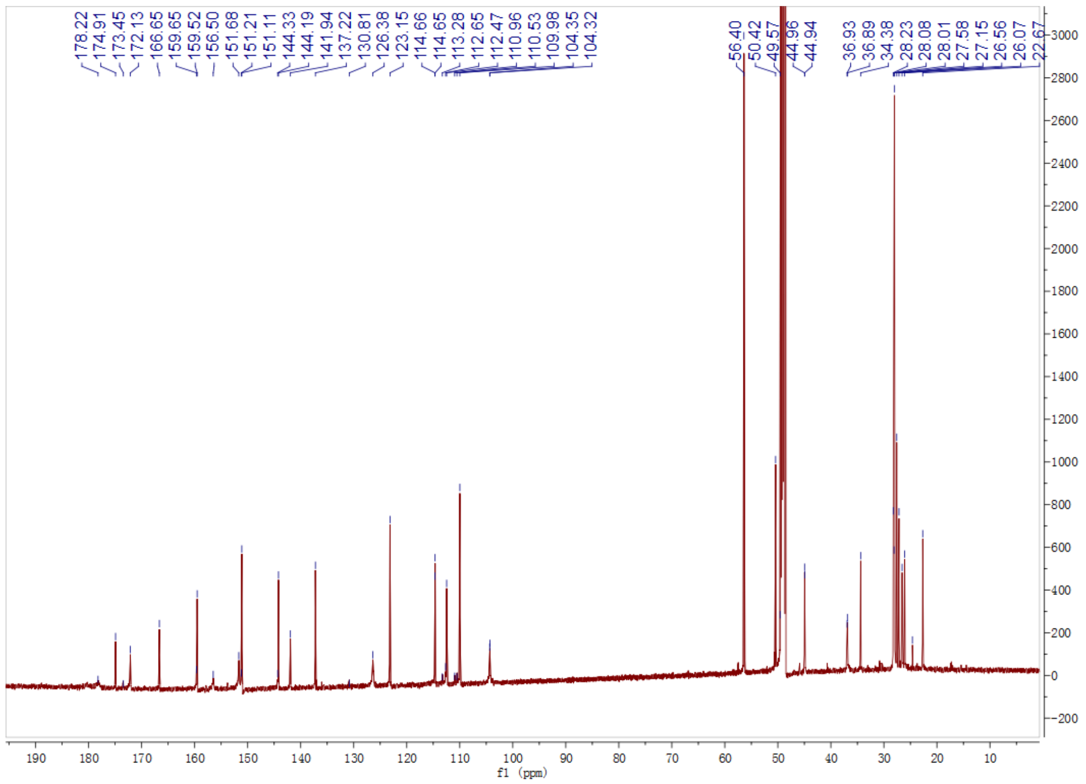

Fig. 15 ^{13}C-NMR spectrum of compound G5

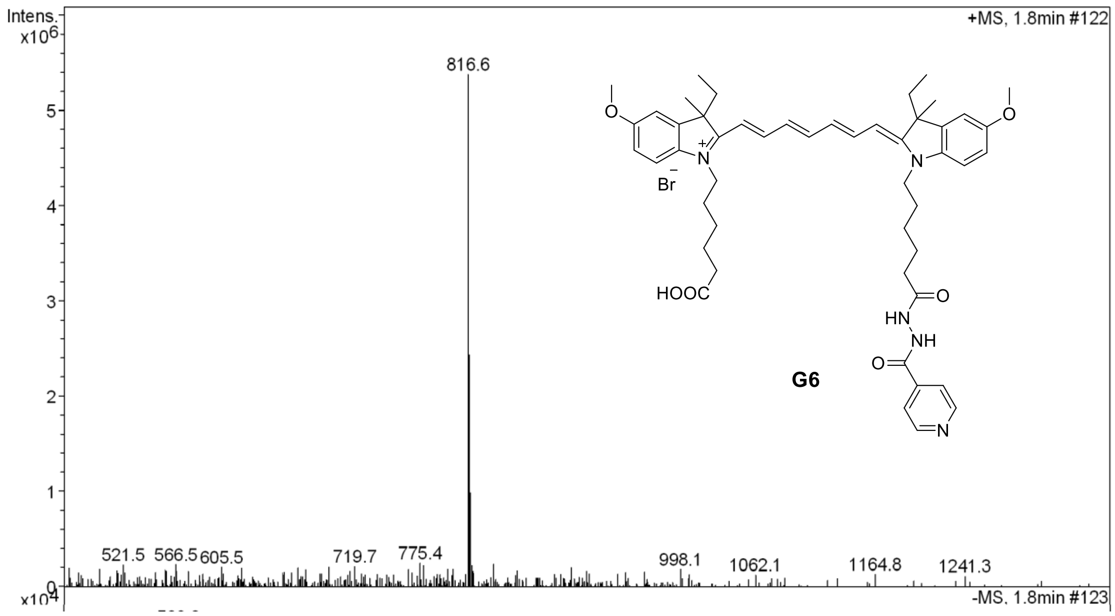

Fig. 16 MS spectrum of compound G6

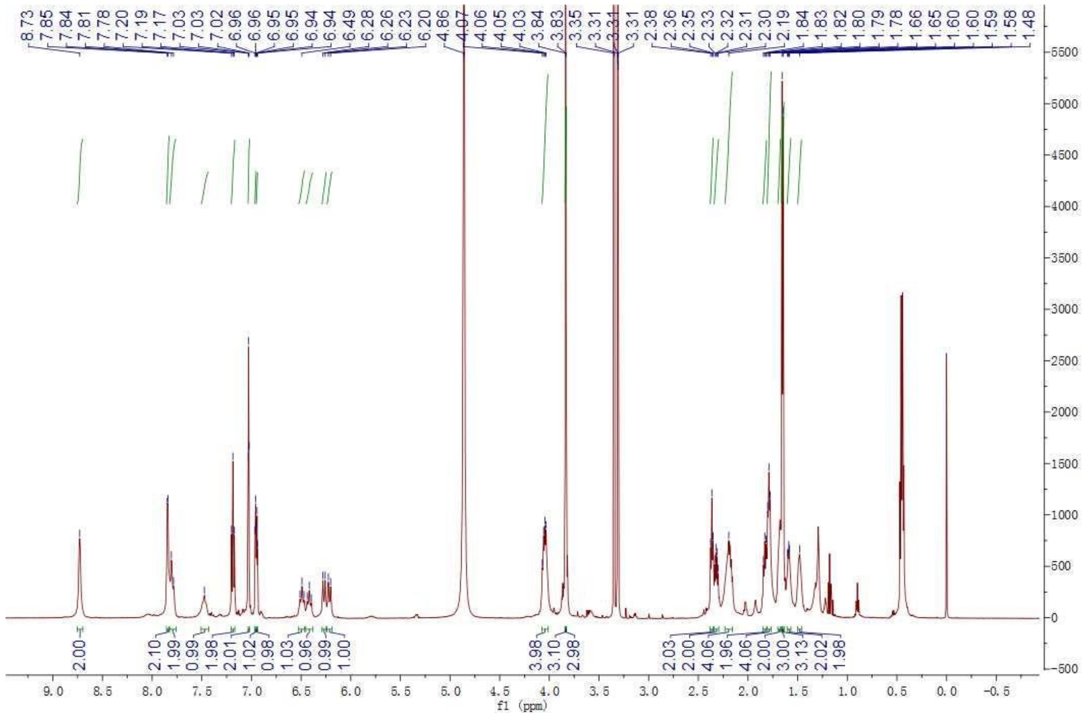

Fig. 17 ^{1}H-NMR spectrum of compound G6

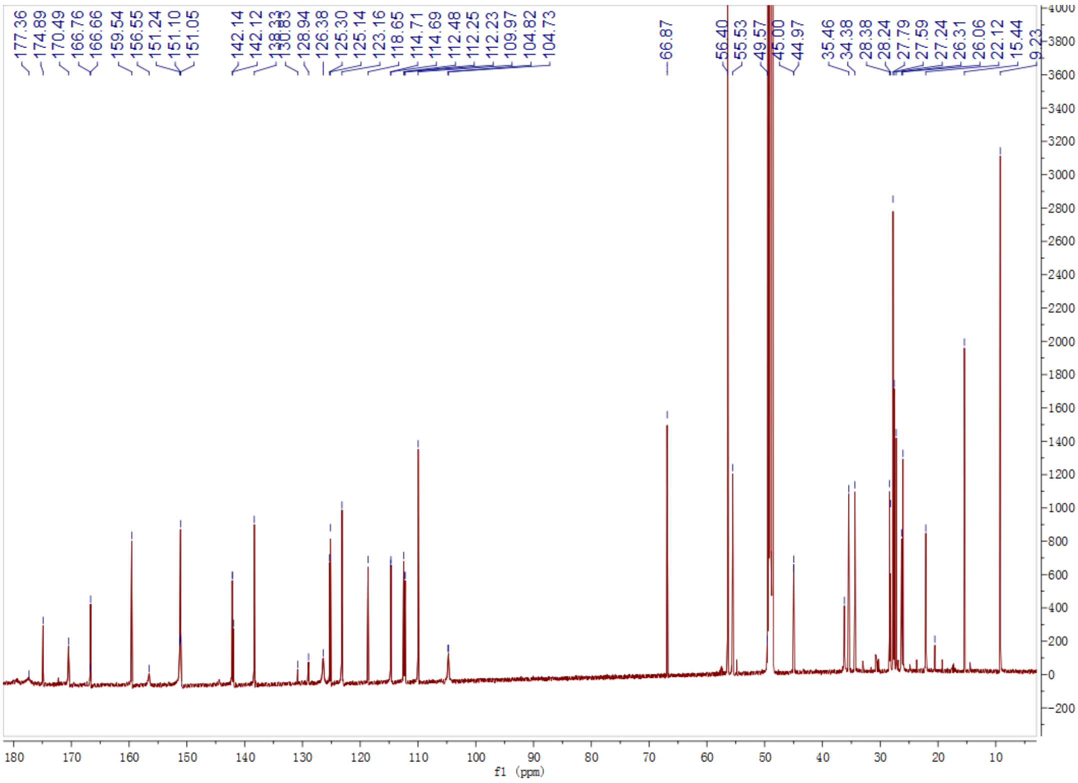

Fig. 18 ^{13}C-NMR spectrum of compound G6

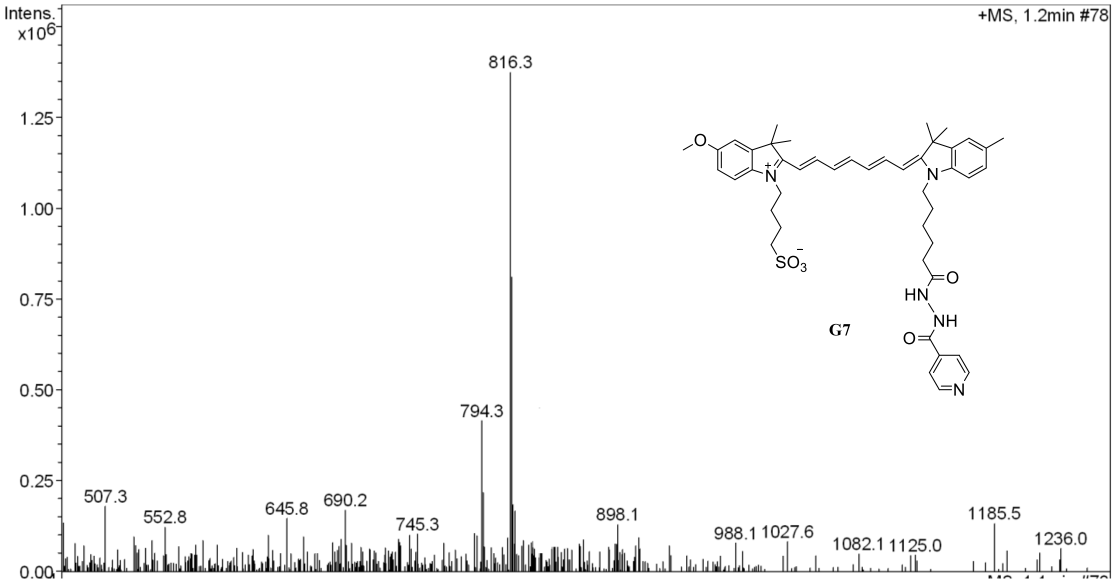

Fig. 19 MS spectrum of compound G7

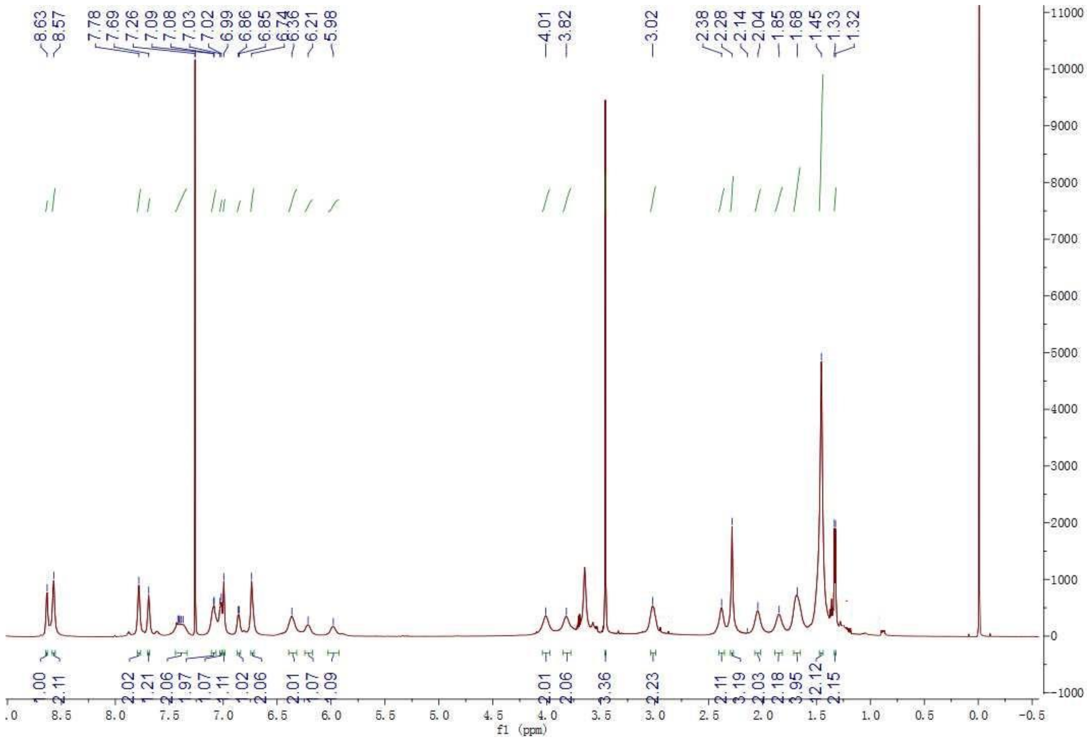

Fig. 20 ^{1}H-NMR spectrum of compound G7

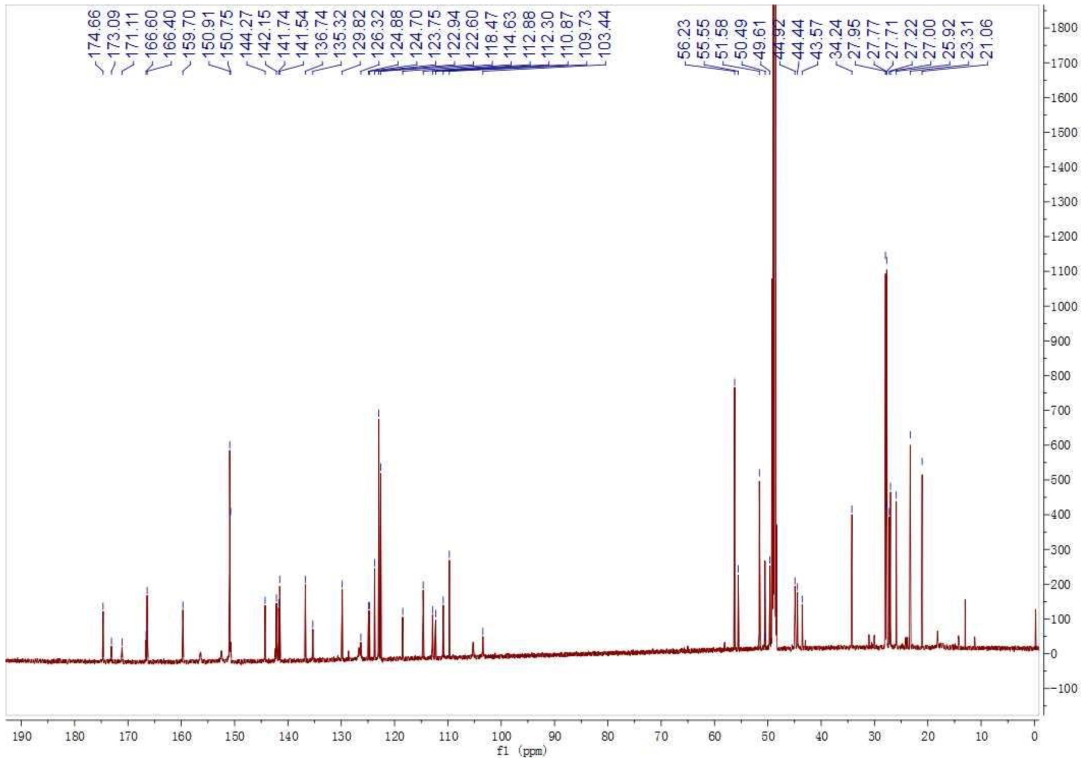

Fig. 21 ^{13}C-NMR spectrum of compound G7

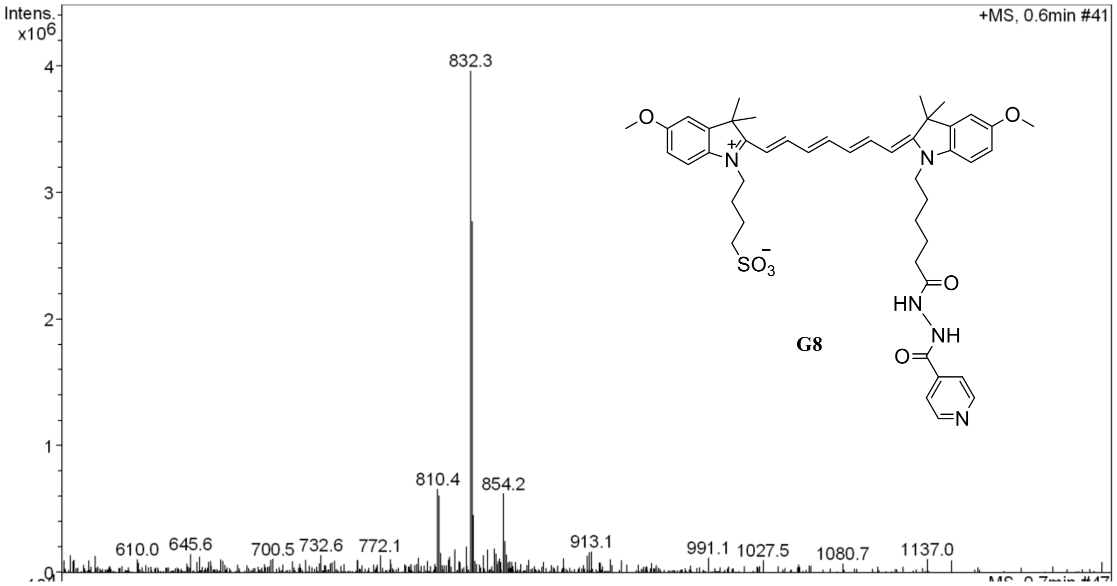

Fig. 22 MS spectrum of compound G8

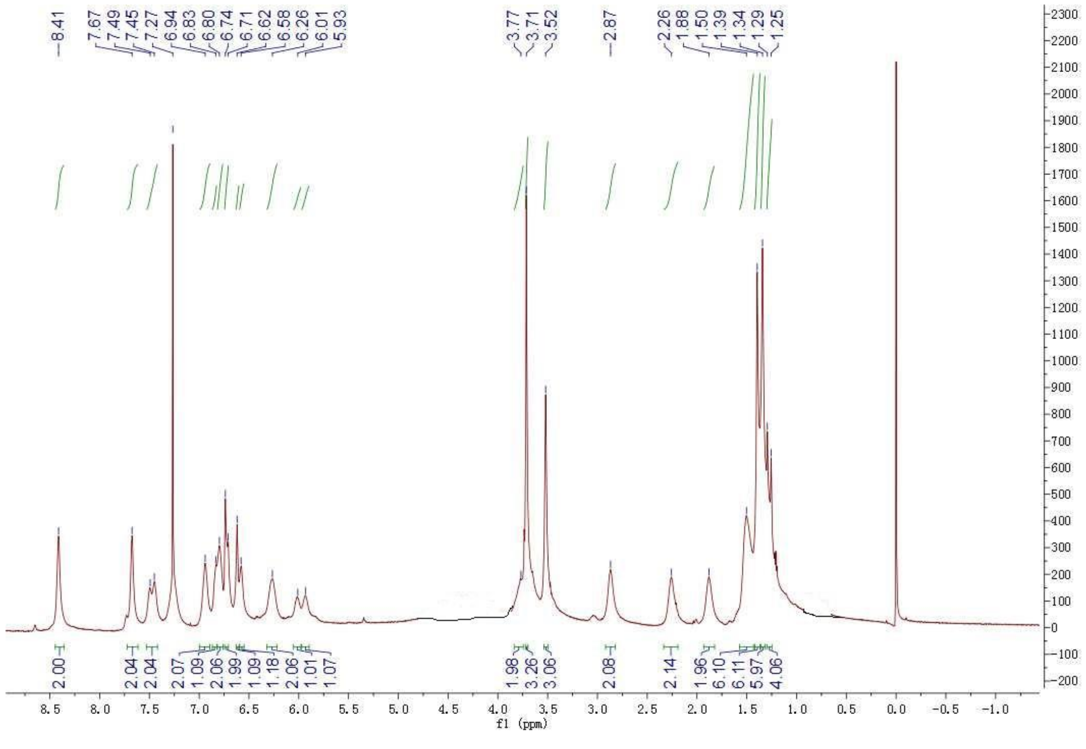

Fig. 23 ^{1}H-NMR spectrum of compound G8

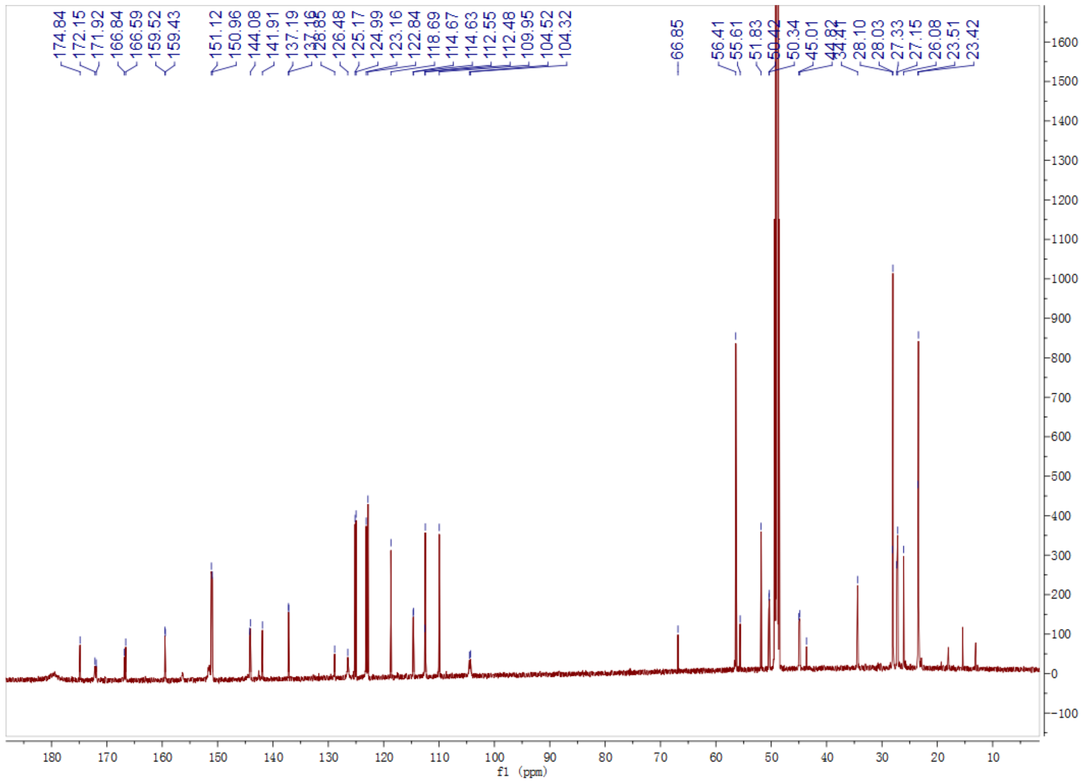

Fig. 24 ^{13}C-NMR spectrum of compound G8

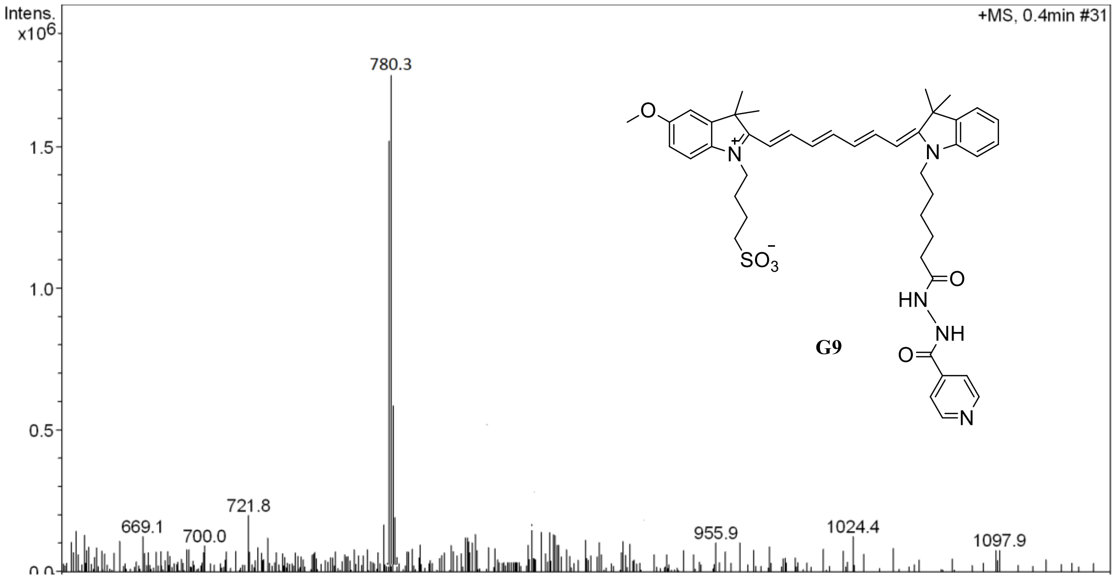

Fig. 25 MS spectrum of compound G9

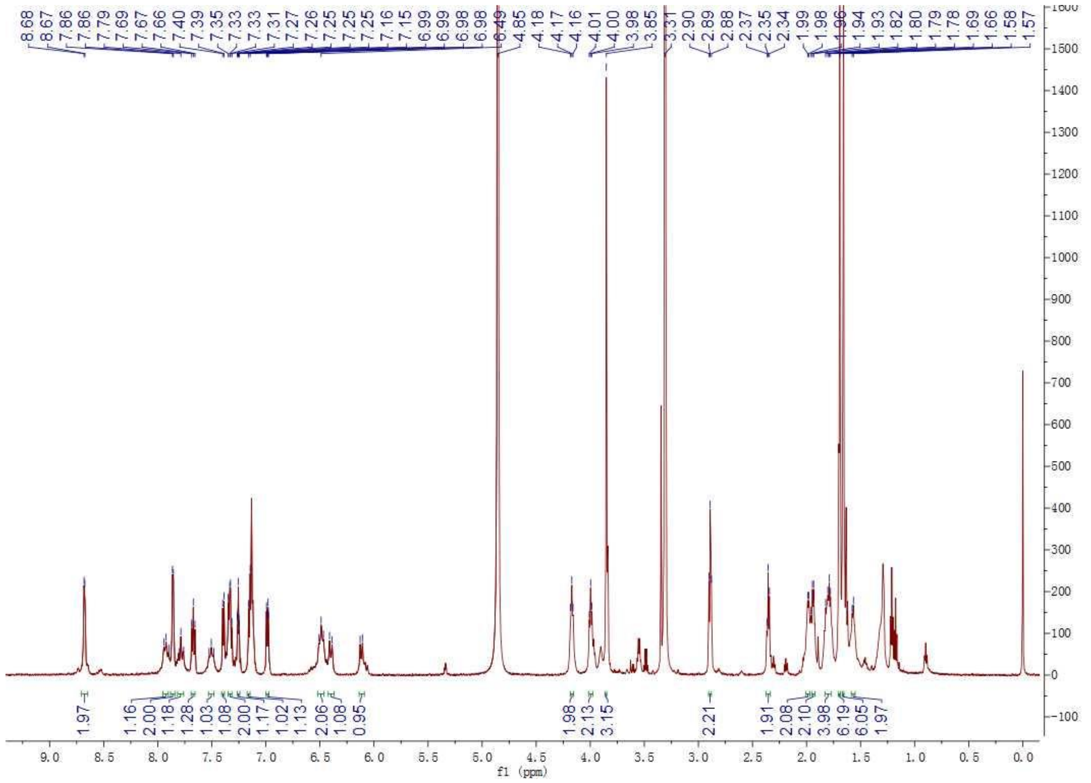

Fig. 26 ^{1}H-NMR spectrum of compound G9

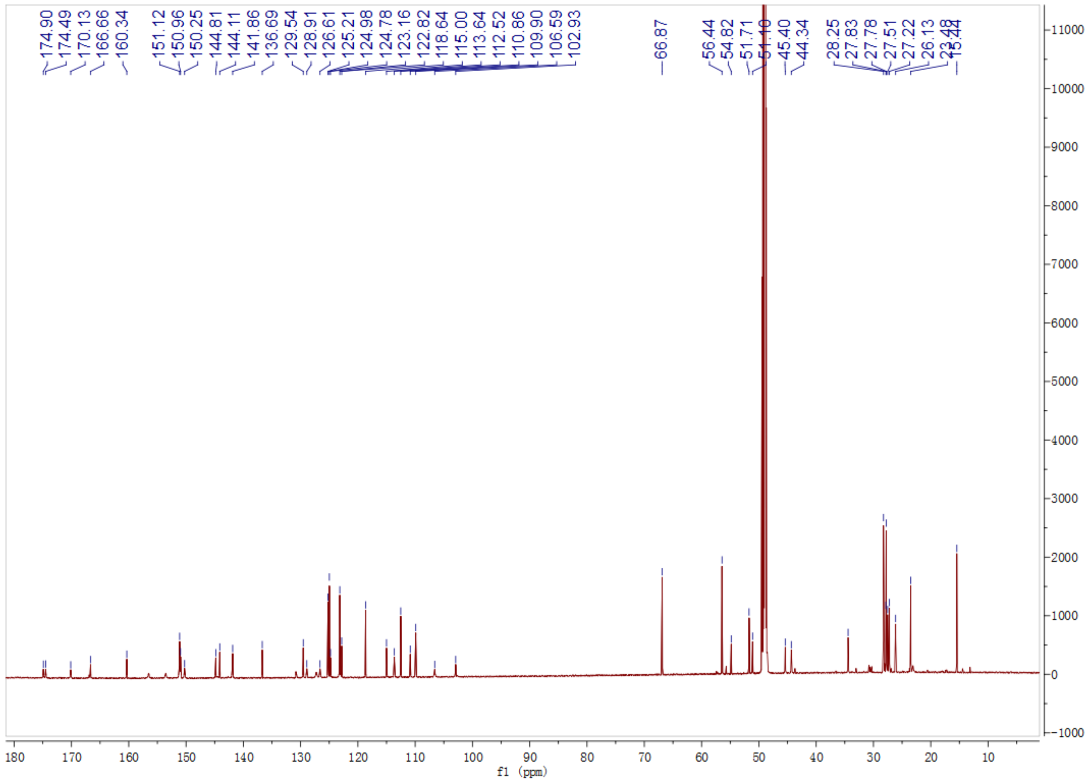

Fig. 27 ^{13}C-NMR spectrum of compound G9

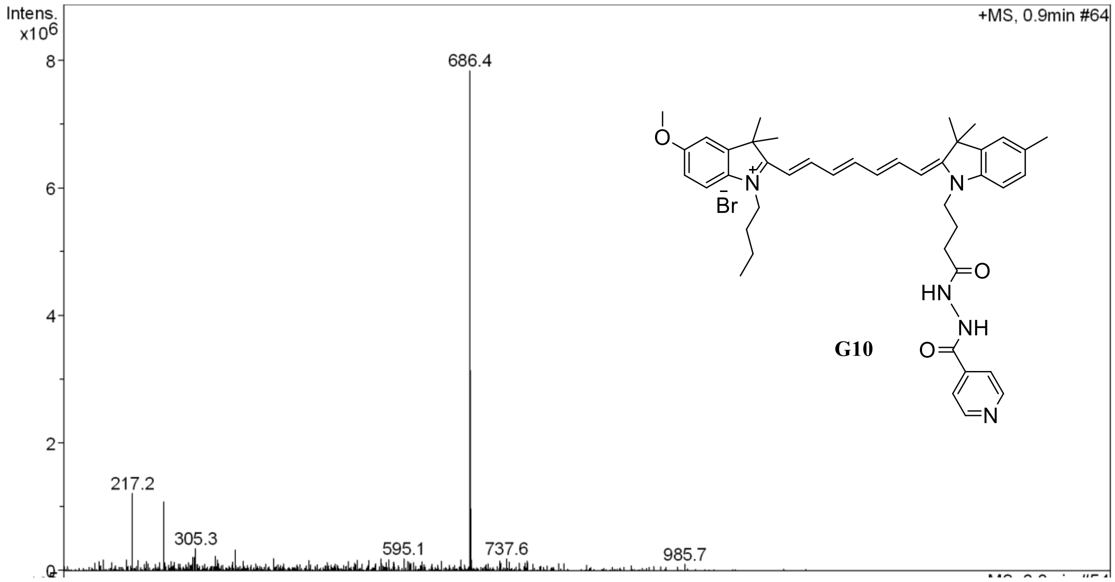

Fig. 28 MS spectrum of compound G10

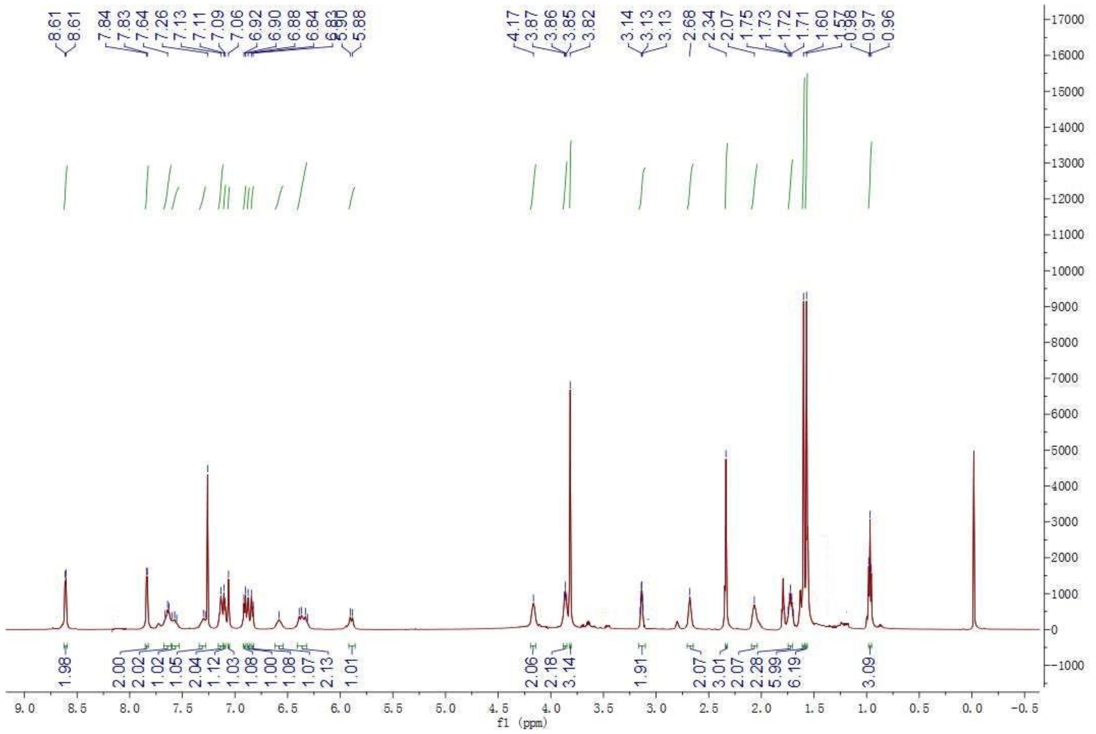

Fig. 29 ^{1}H-NMR spectrum of compound G10

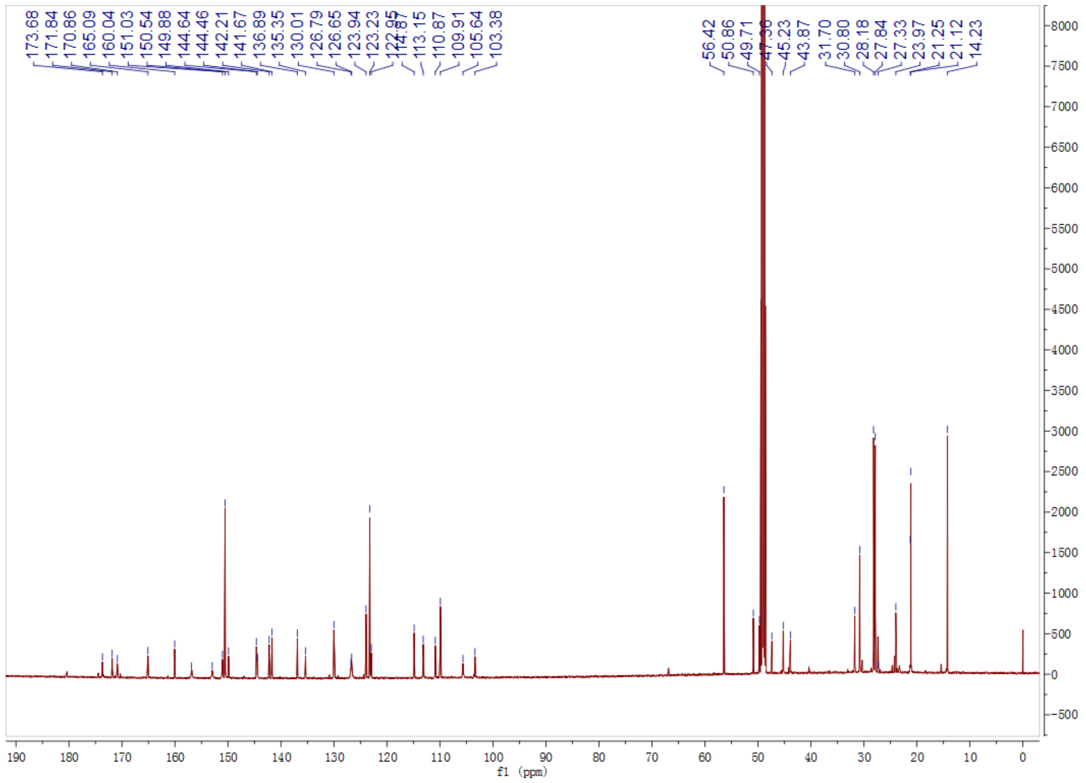

Fig. 30 ^{13}C-NMR spectrum of compound G10

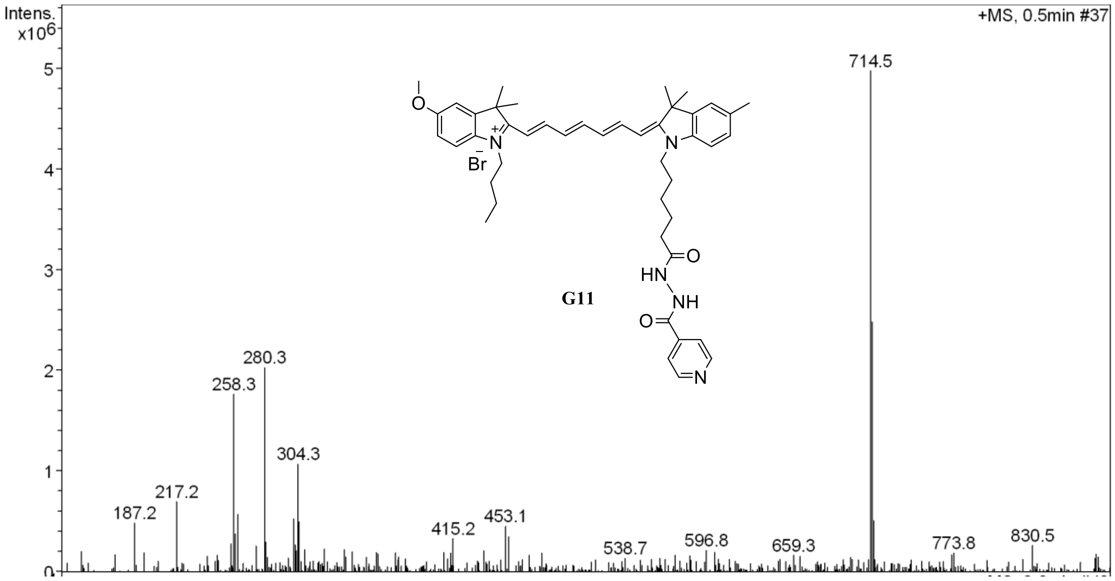

Fig. 31 MS spectrum of compound G11

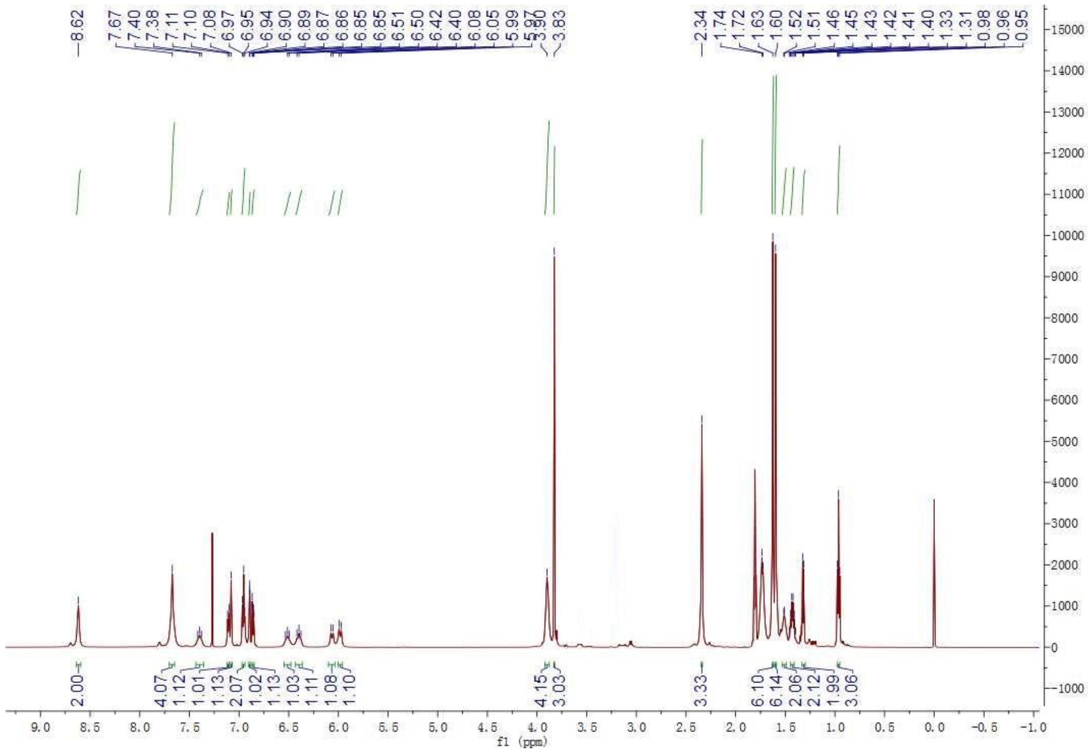

Fig. 32 ^{1}H-NMR spectrum of compound G11

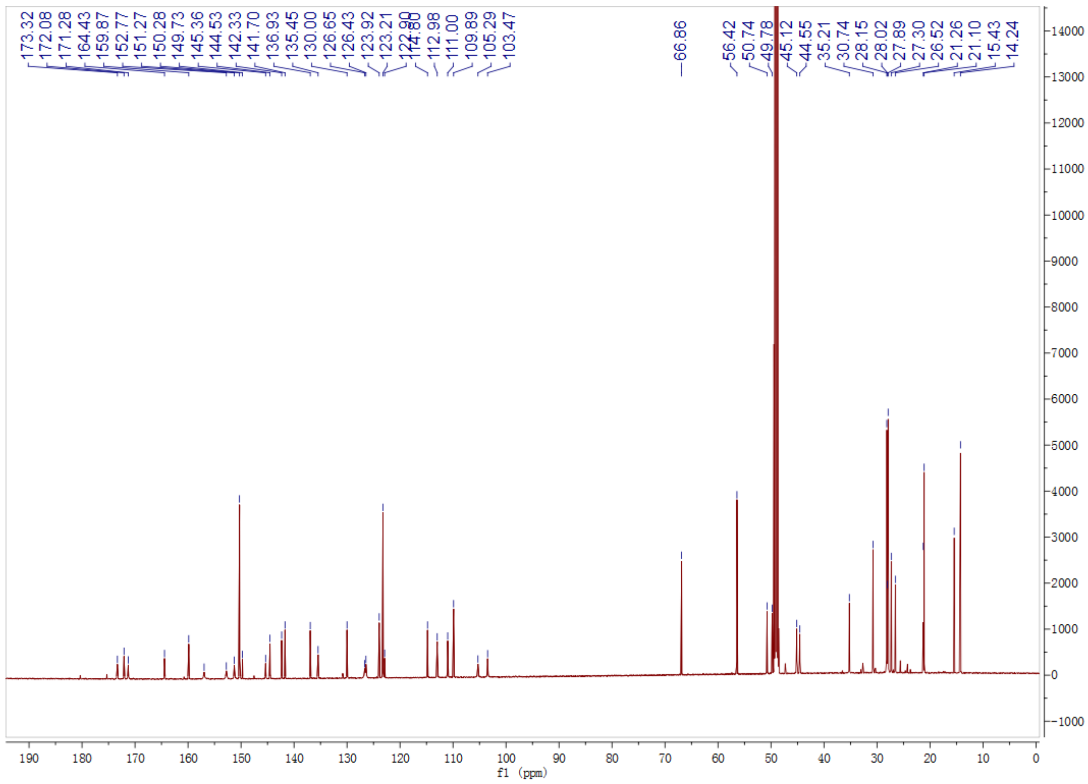

Fig. 33 ^{13}C-NMR spectrum of compound G11

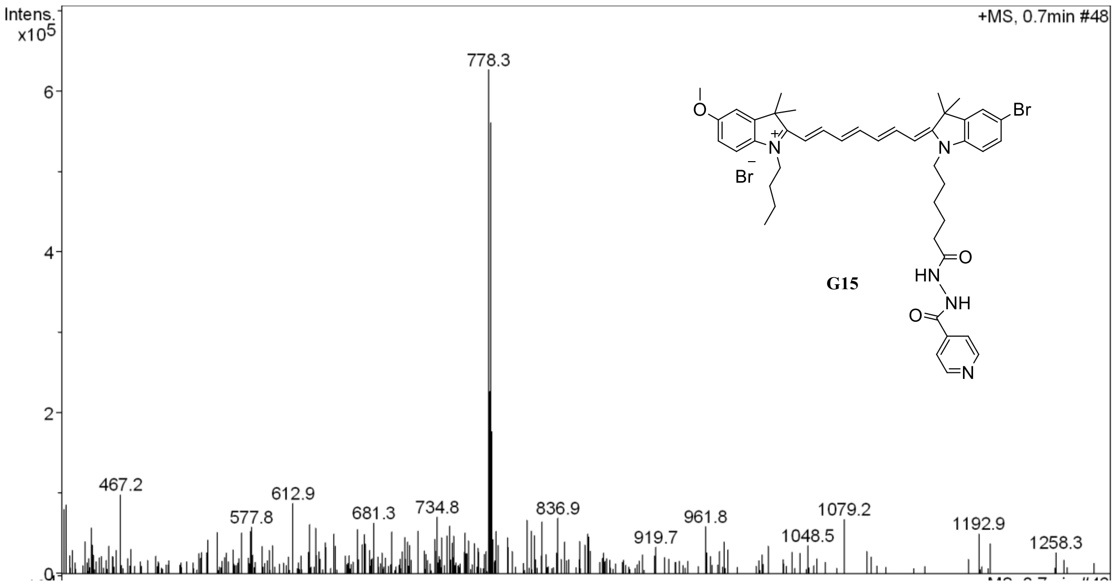

Fig. 34 MS spectrum of compound G12

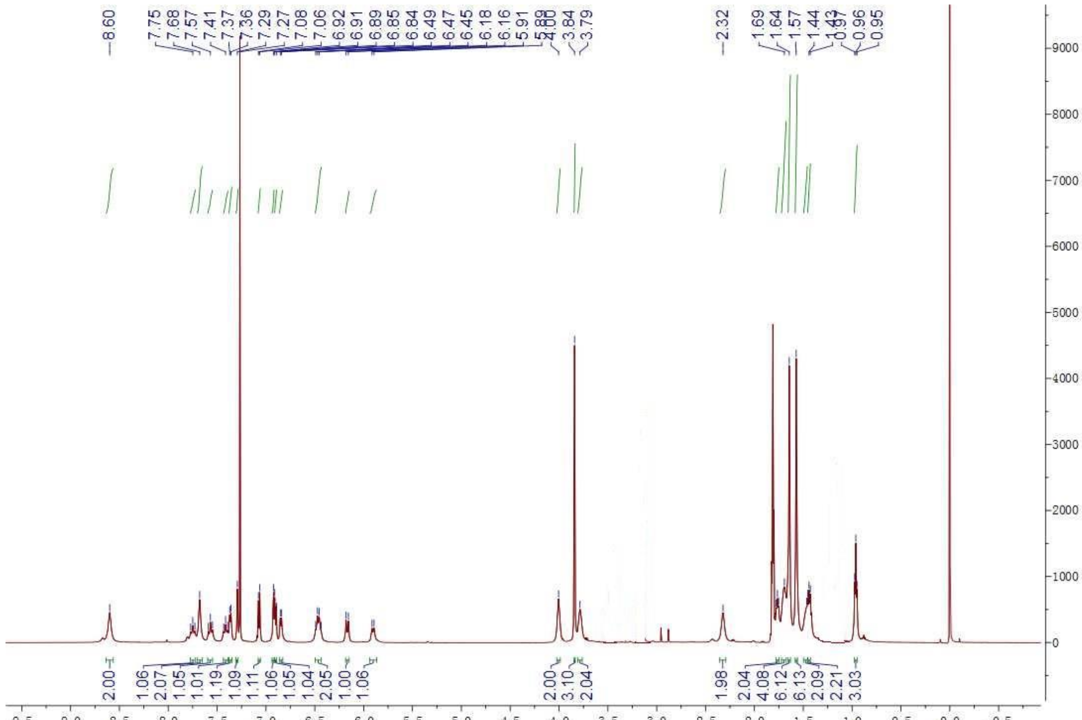

Fig. 35 ^{1}H-NMR spectrum of compound G12

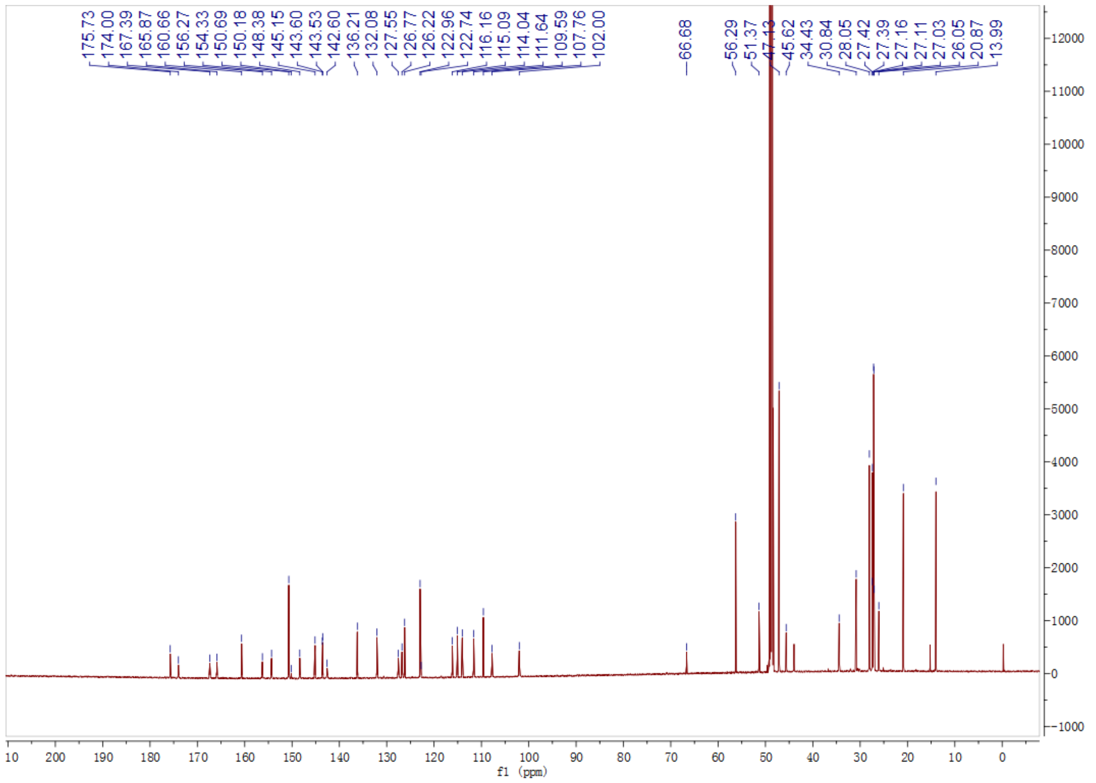

Fig. 36 ^{13}C-NMR spectrum of compound G12

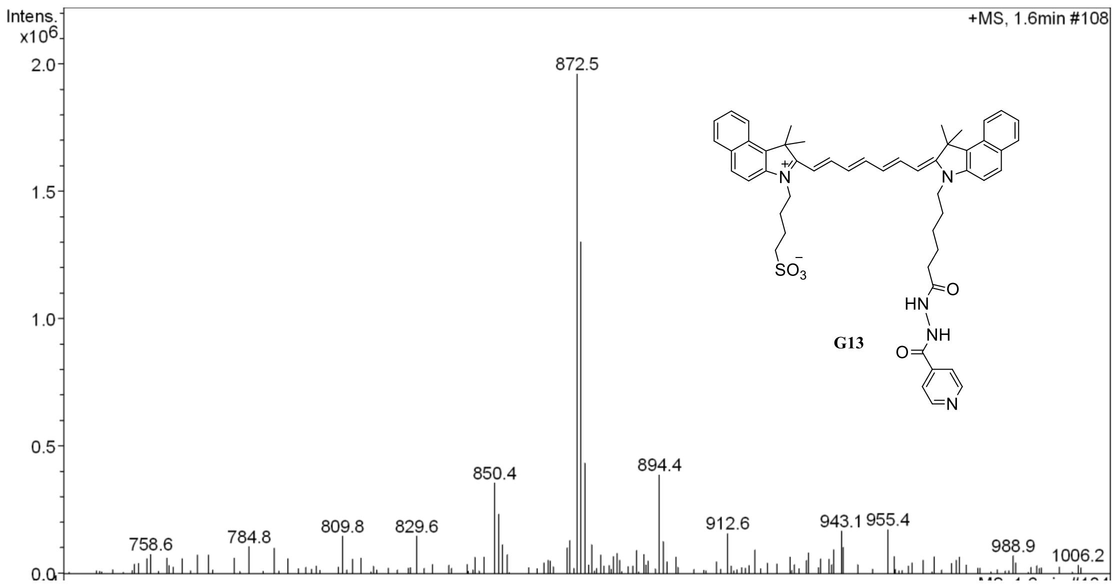

Fig. 37 MS spectrum of compound G13

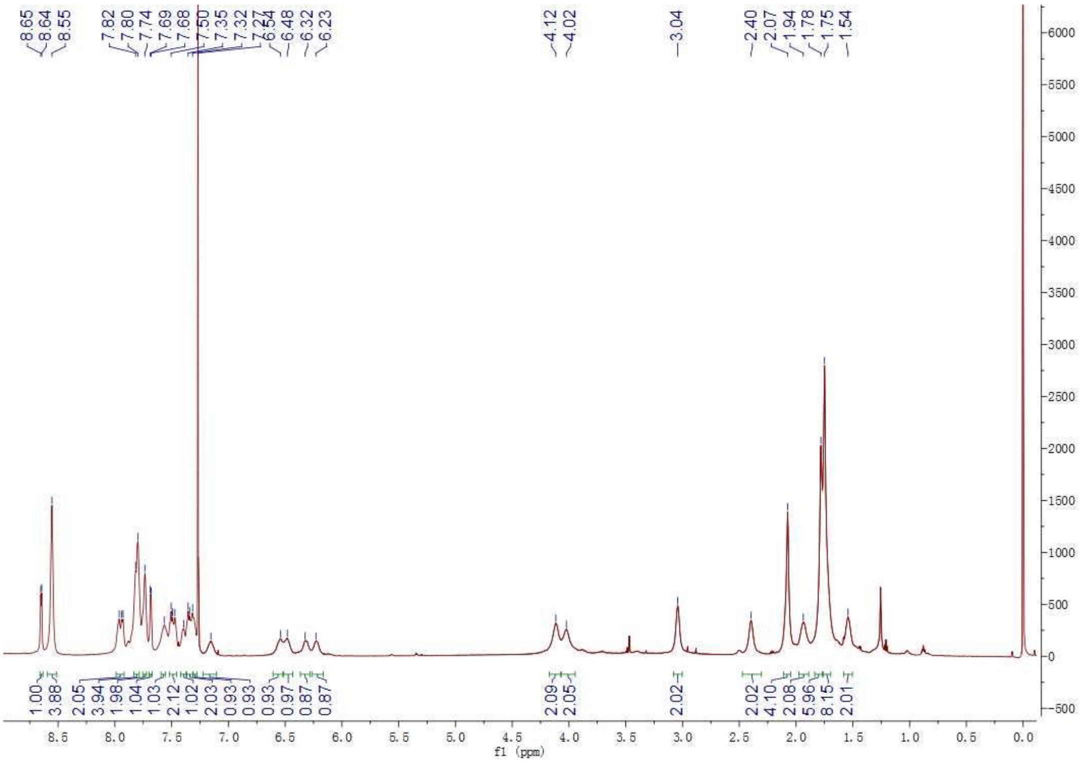

Fig. 38 ^{1}H-NMR spectrum of compound G13

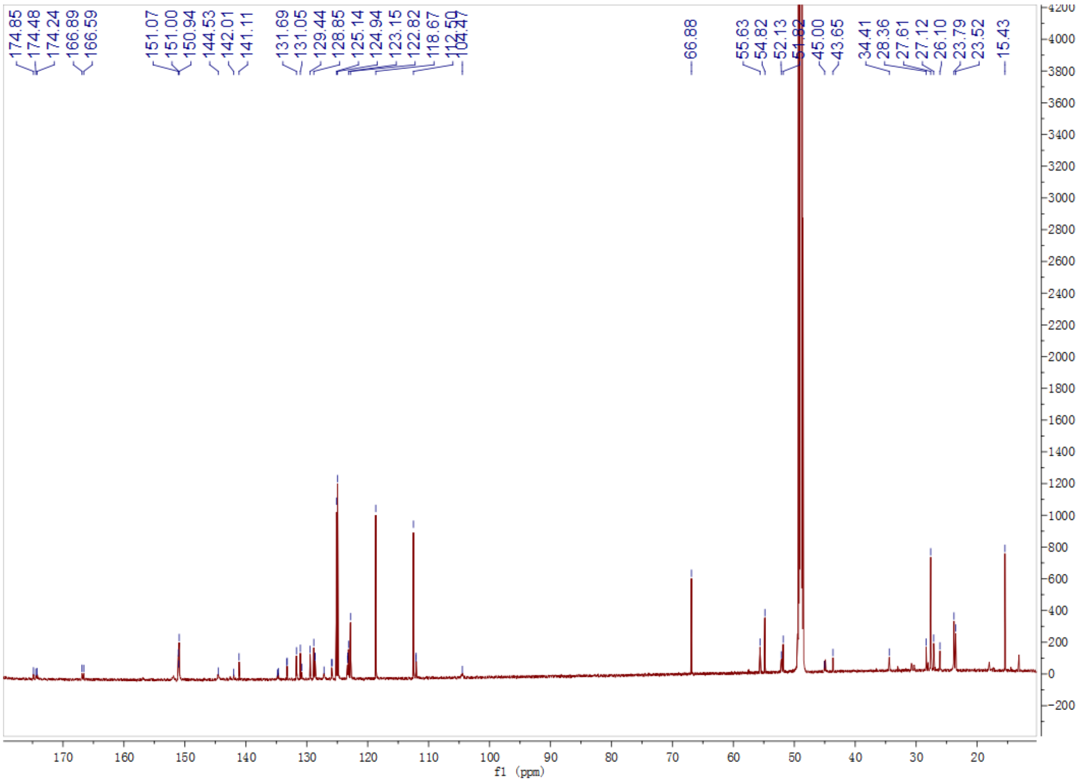

Fig. 39 ^{13}C-NMR spectrum of compound G13
